# Falsely Elevated Tacrolimus (FK506) Trough Levels in a Liver Transplant Recipient

**DOI:** 10.7759/cureus.54548

**Published:** 2024-02-20

**Authors:** Noemi Garg, James Mo, Mary G Fitzmaurice, Sarah Warnke, Syed-Mohammed Jafri

**Affiliations:** 1 Internal Medicine, Wayne State University School of Medicine, Detroit, USA; 2 Gastroenterology, Henry Ford Health System, Detroit, USA; 3 Gastroenterology and Hepatology, Henry Ford Health System, Detroit, USA

**Keywords:** immunosuppression therapy, trough concentration, lc-ms/ms, orthotopic liver transplantation, post liver transplant tacrolimus toxicity

## Abstract

Antibody-conjugated magnetic immunoassay (ACMIA) for tacrolimus (FK506) may detect falsely elevated tacrolimus trough levels, a commonly underreported event. We report a case of falsely elevated whole-blood tacrolimus levels in a patient post-orthotopic liver transplantation. A 71-year-old male patient underwent liver transplantation in 2012. Post-transplantation, the patient was immediately started on tacrolimus for maintenance immunosuppression. His most recent dose was 0.5 mg four times weekly. During monitoring, trough levels were at 25.9 ng/mL using ACMIA. After this result, a decision was made to hold tacrolimus. After holding tacrolimus for seven days, detected trough levels were still continually greater than 20 ng/mL. Upon suspicion of falsely elevated results, liquid chromatography with mass spectroscopy (LC-MS) was used to check tacrolimus trough levels. Results showed normal trough levels of 7.6 ng/mL. Because of its narrow therapeutic window, tacrolimus levels need to be carefully monitored throughout treatment. When high tacrolimus levels are detected using ACMIA without a correlating clinical scenario, trough levels should be re-confirmed using LC-MS to prevent clinical decisions from being made based on falsely elevated results.

## Introduction

Tacrolimus (FK506) is a common immunosuppressant frequently used post-solid organ transplantation to prevent rejection [1]. It is a calcineurin inhibitor (CNI) that primarily exerts its immunosuppressive effects by impairing the gene expression of its target cells, specifically interleukin-2 (IL-2) gene transcription. Tacrolimus binds to FK506, an immunophilin. This complex binds to calcineurin, resulting in its inhibition [2]. Tacrolimus is a highly efficacious drug in preventing transplant rejection. Two randomized trials concluded that the incidence of acute rejection was significantly lower, and the projected graft half-life was longer in patients treated with tacrolimus-based immunosuppression as compared to cyclosporine [3].

However, tacrolimus has many side effects. Tacrolimus has been shown to be nephrotoxic through a variety of mechanisms, with 18% to 42% of liver transplant patients and 17% to 44% of kidney transplant patients experiencing nephrotoxicity from tacrolimus [4]. Tacrolimus nephrotoxicity can result in varying morphologic changes such as acute tubular necrosis, tubular calcifications, and diffuse interstitial fibrosis [1,4]. The clinical presentation of acute tacrolimus nephrotoxicity, either from high trough levels or chronic use of the drug, is defined as a rise in blood urea or serum creatinine levels, not from a secondary cause. This makes the diagnosis of tacrolimus nephrotoxicity one of exclusion, and a definitive diagnosis can be made when there is a decrease in serum creatinine levels following a reduction in tacrolimus dosing [4]. However, chronic tacrolimus nephrotoxicity may be irreversible. The complete mechanisms by which tacrolimus exerts its nephrotoxic effects are still unclear. Additionally, there are also neurotoxic effects associated with tacrolimus. Twenty-five percent to 31% of patients on tacrolimus have had some form of neurotoxic event [5]. Approximately 20% of patients who receive tacrolimus have experienced mild neurotoxicity, including tremors, insomnia, headache, vertigo, dysesthesia, photophobia, and mood disturbance.

The pharmacokinetics of tacrolimus are highly variable. Due to its side effects and potential toxicities, careful therapeutic drug monitoring (TDM) of tacrolimus is imperative. Tacrolimus has a narrow therapeutic window with a target trough concentration range of 6-10 ng/mL in the setting of triple maintenance immunosuppression therapy post-liver transplantation. Because of its narrow therapeutic window and risk of toxicities, tacrolimus needs to be carefully studied and monitored to ensure its safe and effective use in the clinical setting.

Currently, the most common method of measuring whole blood tacrolimus levels is using affinity column-mediated immunoassay (ACMIA). ACMIA is an enzyme immunoassay that measures the color reaction between an antigen and an antibody. The assay is commonly used because there is no need to pretreat samples, reducing assay time [6]. However, there have been several reported cases of falsely elevated tacrolimus levels post-transplant, hypothesized to be due to circulating endogenous heterophile antibodies binding to the reagent, animal antibodies, used in the assay [6-8]. Liquid chromatography and tandem mass spectrometry (LC-MS) is considered the gold standard for monitoring tacrolimus levels due to its selectivity, sensitivity, and specificity [9-11]. However, LC-MS is not often used because it is time-intensive and more expensive. However, there have been improvements to LC-MS in recent years, allowing it to be employed for routine use in the clinical setting. However, it still has its limitations. Improvements to LC-MS are being developed to warrant the widespread use of LC-MS in the measurement of tacrolimus levels, especially in cases of detected elevations using ACIMA.

Here, we report a case of a patient with falsely elevated tacrolimus levels using ACMIA post-orthotopic liver transplantation who upon subsequent retesting using LC-MS was found to have therapeutic trough levels of tacrolimus.

This article was previously presented as a meeting abstract at the American College of Gastroenterology (ACG) 2021 Annual Scientific Meeting from October 22 to 27, 2021 [12].

## Case presentation

**Figure 1:**
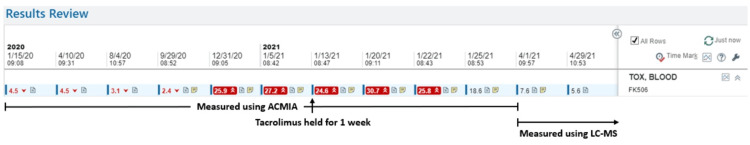
Patient tacrolimus trough levels with the timeline of events

We report a case of a 71-year-old male patient with a history of hepatitis C cirrhosis who underwent orthotopic liver transplantation in 2012. He presented to our institution for an annual routine wellness visit post-transplantation. For several years, the patient had been maintained on 0.5 mg four times weekly of immediate-release tacrolimus with trough levels being routinely checked. The patient was within the goal trough level until December 2020 when his trough levels were found to be supratherapeutic at 25.9 ng/mL (goal: 6-8 ng/mL) as detected by ACMIA. He had never had any supratherapeutic levels before this result. His most recent trough levels prior to this were 2.4 ng/mL. The patient stated that tacrolimus was not taken before his lab work and he did not start any new medications or supplements that may contribute to supratherapeutic levels of tacrolimus on blood draw. The patient also did not consume any grapefruit or pomegranate juice or have any significant dietary changes. He did not present with any usual symptoms of tacrolimus toxicity such as any recent seizure history, tremors, hypertension, elevated serum creatinine or potassium, or confusion. However, due to the risk of developing nephrotoxicity, the decision was made to hold his tacrolimus for one week and repeat lab results. One week later, his trough levels for tacrolimus were checked again using ACMIA and were still greater than 20 ng/mL or significantly elevated from baseline. The patient was still without any clinically significant toxicity symptoms. Liver function tests were also at baseline and there were no signs of graft rejection. Due to the discrepancy between the trough levels and the patient’s clinical presentation, there was suspicion of false elevations with ACMIA. The patient was subsequently retested using LC-MS. Results showed normal trough levels of 7.6 ng/mL, invalidating the previous ACMIA results. Continued measurements later were done using LC-MS. His subsequent trough levels after that were 5.6 ng/mL while on tacrolimus (Figure 1). Since then, all trough levels have been within normal limits and never reached supratherapeutic levels using LC-MS, and the patient did not develop any adverse reactions from continuing his previous home dose.

## Discussion

Tacrolimus is a widely used drug for immunosuppression post-transplant. It has a narrow therapeutic window and thus the trough levels of tacrolimus are monitored closely through regular lab work. Two methods exist to measure trough levels: ACMIA and LC-MS. ACMIA is the more popular, quicker, and cheaper method of detection. However, it has been reported to show erroneous supratherapeutic tacrolimus trough levels due to cross-reactions with drug metabolites during testing.

Unnecessary changes to immunosuppressive therapy and graft rejection are commonly due to falsely elevated results falsely elevated trough levels using ACMIA. This can be caused by anti-β-galactosidase antibodies. One case study presented a post-kidney transplant patient on tacrolimus having three- to seven-fold higher tacrolimus levels when measured using ACMIA compared to enzyme-multiplied immunoassay technique (EMIT) and LC-MS due to an unidentified antibody [13]. Another case study reported falsely increased tacrolimus concentrations using ACMIA in a post-liver transplant patient, possibly due to heterophilic antibodies [14]. Finally, a third case study described a patient as having elevated tacrolimus levels measured using ACMIA despite being started on a low dosage [15]. Tacrolimus doses were subsequently adjusted, but soon after, the patient’s serum creatinine began to rise. An allograft biopsy revealed acute T cell-mediated rejection. ACMIA still revealed elevated tacrolimus levels. However, when the patient’s tacrolimus levels were reevaluated using EMIT, tacrolimus levels were below detection limits. Thus, the rejection was suspected to be due to low levels of tacrolimus. After tacrolimus dosing was increased and levels were monitored using EMIT, kidney function was stable, tacrolimus levels were stable, and there was no rejection five years later [16]. This last case demonstrates the serious consequences of falsely elevated tacrolimus levels. These inaccurate measurements can lead to misguided decreasing of tacrolimus dosing, ultimately resulting in solid-organ rejection. Anti-β-galactosidase antibodies, anti-tacrolimus monoclonal antibodies, human anti-mouse antibodies, and rheumatoid factor have all been reported to falsely elevate tacrolimus levels in ACMIA and other immunoassays. In addition, immunotherapy, vaccinations, polyclonal gammopathy, and blood transfusions can also result in interference with circulating antibodies. Still, there are reported cases of unidentified antibodies having caused falsely elevated tacrolimus levels in transplant patients [13,15-17]. The absence of pre-treatment in ACMIA, which is performed in other immunoassays to remove such antibodies, makes this assay more susceptible to erroneous measurements [18]. Thus, because tacrolimus is mainly distributed in erythrocytes, one possible solution is to compare tacrolimus levels in whole blood, plasma, and washed erythrocytes to determine whether falsely elevated tacrolimus levels using ACMIA are due to interfering antibodies [15].

In this case review, the patient was found to have falsely elevated levels of tacrolimus, which led to holding doses for one week. Low concentrations of tacrolimus in the blood can be associated with transplant rejection and lead to severe consequences for patients. Supratherapeutic levels can cause severe neurotoxicity and nephrotoxicity, amongst other side effects. An improved strategy to avoid unnecessary dose reductions or other changes in immunosuppressive therapy due to false levels is to utilize a more reliable, efficacious method of detecting trough levels in patients. LC-MS does not have the disadvantages of immunoassay while providing a superior detection of tacrolimus without the disadvantages of the immunoassay. However, LC-MS may be more time-consuming and expensive. Several methods using LC-MS have been described but most require online extraction procedures. These procedures require an additional pump, switch valve, and trapping column, making this technique more technically difficult. In addition, different LC-MS setups are required to analyze different immunosuppressants.

## Conclusions

Tacrolimus is a commonly used immunosuppressant post-orthotopic liver transplantation that has a narrow therapeutic window. When high tacrolimus levels are detected using ACMIA without a correlating clinical scenario, trough levels should be re-confirmed using LC-MS. This is necessary to prevent clinical decisions from being made based on inaccurate results. Widespread use of the LC-MS technique to avoid false elevations in trough levels and subsequent inappropriate dose reductions should be considered.
